# A ratiometric fluorescent probe monitoring lipid peroxidation within lipid droplets in foam cells

**DOI:** 10.1002/smo2.70015

**Published:** 2025-08-19

**Authors:** Dingxuan Li, Caiwei Lu, Shulan Sun, Xiaoxi Li, Yi Xiao, Xinfu Zhang

**Affiliations:** ^1^ State Key Laboratory of Fine Chemicals Frontiers Science Center for Smart Materials Oriented Chemical Engineering Dalian University of Technology Dalian China; ^2^ Central Laboratory Cancer Hospital of Dalian University of Technology (Liaoning Cancer Hospital & Institute) Dalian China

**Keywords:** foam cells, lipid droplets, lipid peroxidation, ratiometric probe

## Abstract

Lipid peroxidation (LPO) in foam cells is crucial for regulating atherosclerosis progression. It correlates with lipid uptake and the state of lipid droplets. In this study, we report a lipid droplet‐targeted fluorescent LPO probe, Ld‐LPO. It selectively responds to LPO, resulting in a significant fluorescence shift from 590 to 525 nm, enabling a ratiometric imaging of LPO in lipid droplets. Ld‐LPO traces lipid droplets in foam cells, revealing a correlation between LPO and lysosomal engulfment. We found that lipid droplets engulfed by lysosomes exhibit higher LPO, attributed to low‐density lipoprotein accumulation in lysosomes. Furthermore, Ld‐LPO is compatible with dual‐color flow cytometry, facilitating high‐throughput analysis of LPO in foam cells.

## Graphical abstract

A lipid droplet‐targeted LPO probe was developed. It localizes specifically within lipid droplets and shows high sensitivity toward LPO. Using Ld‐LPO, a significant LPO level in lipid droplets that is synchronous with lipid droplets autophagy was observed.

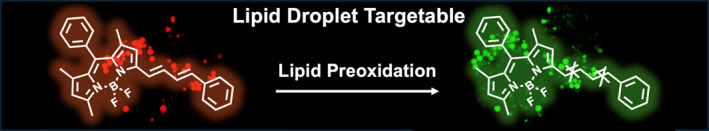

## 1 INTRODUCTION

Lipid peroxidation (LPO) stands as a primary pathological mechanism in numerous diseases, including cancer, neurodegenerative disorders, and atherosclerosis.[^1^, ^2^, ^3^, ^4^, ^5^, ^6^, ^7^, ^8^] LPO essentially involves the oxidation of polyunsaturated fatty acids (PUFAs) mediated by reactive oxygen species (ROS).[^9^, ^10^] While ROS typically initiates LPO, its pathogenic effects extend beyond the direct oxidative damage to cell membrane lipids caused by ROS. The significant impact of LPO arises from chain propagation, which continuously releases unstable lipid peroxides that further decompose into various reactive and diffusible small molecules, such as malondialdehyde. Reactive aldehydes can alter nucleic acids and proteins, while other bioactive products may influence gene transcription and signal transduction among other processes.

In eukaryotic cells, each organelle possesses its functionality^[11]^ and interacts with other organelles to form an intricate network of organelle interactions.[^12^, ^13^, ^14^] Macrophage foam cell formation represents a typical process occurring at atherosclerotic sites, characterized by lysosomal foaminess and elevated intracellular LPO levels, with the foaming process closely associated with lipid uptake.^[15]^ At present, many probes targeting lipid droplets have been reported based on a series of fluorescent dyes such as naphthalimide, NBD, and BODIPY derivatives, such as BODIPY 505/515. On this basis, LD‐LPO has the function of real‐time monitoring of LPO levels in lipid droplets, which has particular significance for the diagnosis of many diseases. Lipid droplets serve as storage sites for neutral lipids and interact with numerous organelles.[^16^, ^17^] Meanwhile, lysosomes, dynamic digestive organelles with an acidic luminal pH, play pivotal roles in endocytosis, autophagy, and phagocytosis pathways.^[18]^ The degradation of lipid droplets via lysosomes is termed lipophagy. In the lipophagy process, excess components and damaged lipid droplets are believed to be transported to lysosomes for degradation and metabolism.[^11^, ^19^] Furthermore, lipophagy is closely associated with certain metabolic disorders and chronic inflammation.^[20]^ Lysosomal expansion is a notable feature of foam cells, characteristic of atherosclerosis. Tracing lipid droplets and monitoring LPO levels are essential for understanding the formation and development of macrophage foam cells, providing deeper insights into the mechanisms underlying disease occurrence. However, whether lysosomes engulf lipid droplets during lysosomal expansion and whether the LPO levels of lipid droplets are affected by lysosomal engulfment remain to be directly evidenced.

**FIGURE 1:**
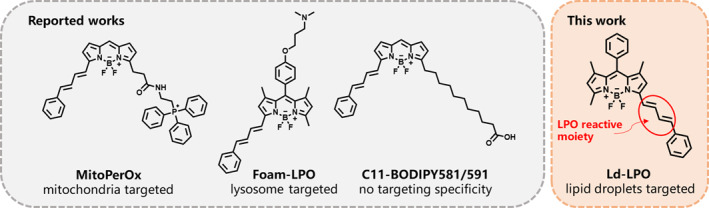
Structures of reported lipid peroxidation (LPO) probes and Ld‐LPO.

To our knowledge, reliable tools for real‐time monitoring of local LPO within lipid droplets are lacking. Although fluorescence imaging is frequently used to assess cellular LPO using probes such as C11‐BODIPY, MitoPerOx, and Foam‐LPO (Figure 1), there is a lack of fluorescent LPO probes specifically targeted to lipid droplets.[^21^, ^22^, ^23^] Therefore, we are dedicated to developing and applying the first lipid droplet‐targeted LPO probe, Ld‐LPO (Figure 1). We developed this fluorescent probe based on diene BODIPY. It is applied to visualize lipid droplets and their lipid peroxides (LPO) through ratiometric imaging and colocalizing imaging with a reported lysosomal probe Lyso‐700.^[24]^

## 2 RESULTS AND DISCUSSION

### 2.1 Design and synthesis of Ld‐LPO

To monitor local LPO within cellular lipid droplets, the fluorescent probe should meet two fundamental requirements: sensitivity to LPO and specific localization within lipid droplets throughout the cellular pathology process. Diene moiety in Ld‐LPO serves as the responsive unit to LPO. When simulating the oxidation reaction between PUFAs in lipids and the conjugated diene moiety, the conjugated length of the fluorophore is shortened. Consequently, Ld‐LPO can respond to LPO through spectral shifts or ratio changes. This concept of using diene‐BODIPY as a signaling unit is derived from previous LPO probes such as C11‐BODIPY, MitoPerOx, and Foam‐LPO.[^25^, ^26^] Additional advantages of this signaling unit include high selectivity for LPO and insensitivity to environmental factors.

**FIGURE 2:**
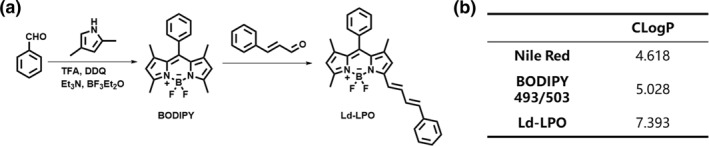
(a) Synthetic route of foam‐LPO. (b) Common lipophilic dyes and their corresponding ClogP values.

Compared with the reported probe C11‐BODIPY581/591, we removed all structural modification groups, resulting in the new probe's high lipophilicity. Ld‐LPO tends to localize within lipid droplets in cells and restores its intense red fluorescence. The ClogP value is the LogP value of a compound predicted by computer simulation, representing the predicted lipophilicity of the compound. Using Chemdraw 20 for the lipophilicity prediction of compound Ld‐LPO, its ClogP value was calculated to be 7.393 (Figure 2b), higher than common lipophilic dyes such as Nile Red, and BODIPY 493/503 (Supporting Information S1 for structure details). This indicates the probe's propensity for staining lipid droplets. Additionally, Ld‐LPO synthesis is straightforward, requiring only a one‐step Knoevenagel condensation reaction (Figure 2a). The starting materials are commercially available reagents, inexpensive, and readily accessible.

### 2.2 Optical properties of Ld‐LPO

**FIGURE 3:**
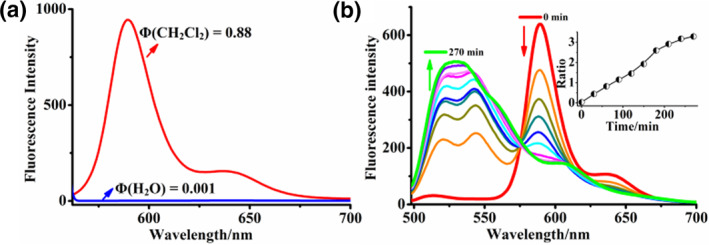
(a) Fluorescent quantum yield of Ld‐LPO in phosphate‐buffered saline (PBS) (pH = 7.2) and CH_2_Cl_2_. (b) Changes in the fluorescence wavelength of Ld‐LPO in CH2Cl2 in the presence of 1 μM hemin and 1 mM CumOOH, excited at 488 nm, and the relationship graph between the fluorescence ratio (525 and 590 nm) and time.

The optical properties study reveals that Ld‐LPO exhibits a high fluorescence quantum yield of up to 0.88 in dichloromethane. In contrast, the probe shows minimal fluorescence in phosphate‐buffered saline (PBS, pH = 7.2), with a quantum yield of 0.001 (Figure 3a). The experiments illustrate the extremely high lipophilicity of Ld‐LPO, evidenced directly by aggregation that quenches its fluorescence in water, thereby reducing background signal interference in the fluorescence imaging of lipid droplets not targeted in the cytoplasm. Subsequently, we validated the material's responsiveness to LPO in solution. As expected, Ld‐LPO displays a spectral shift sensitive to LPO, similar to C11‐BODIPY581/591, MitoPerOx, and Foam‐LPO. Our LPO probe features a fluorescent structure with a conjugated diene linking the BODIPY core and the phenyl moiety. Due to this extended conjugation, the intact Ld‐LPO exhibits bright red fluorescence with maximal emission at 590 nm. Under appropriate induction of ROS, this conjugated diene undergoes oxidation, leading to the degradation of one or both double bonds. Once this occurs, the conjugated system shortens to the BODIPY core, emitting green fluorescence at a shorter wavelength. We simulated LPO in PBS (containing 30% methanol, pH = 5) by adding hemin chloride and CumOOH. This facilitates radical generation from CumOOH in the solution, similar to LPO in living cells.^[27]^ During oxidation, a sustained fluorescence enhancement at 525 nm and a continuous decrease at 590 nm were observed, with the (I_525_/I_590_) ratio changing from 0.03 to 3.28 (Figure 3b). Such a substantial ratio change is desirable for monitoring and imaging cells, enhancing accuracy and reliability in relative quantitative detection. This significant ratio change indicates the suitability of Ld‐LPO for detecting lipid peroxides (LPO) within cells.

### 2.3 Lipid droplet‐targeting of Ld‐LPO in macrophage cells

**FIGURE 4:**
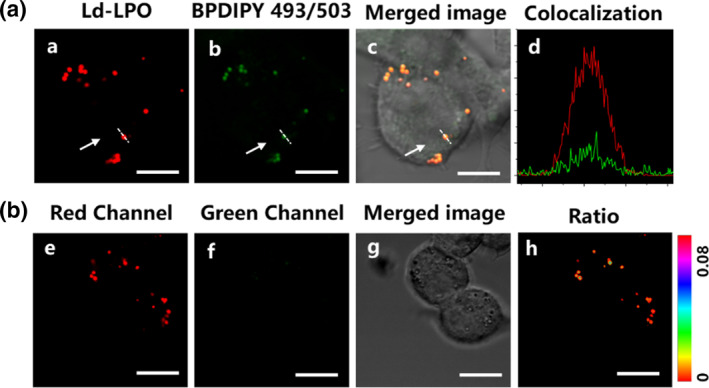
(a) Fluorescence images of Raw264.7 co‐stained by BODIPY493/503 (a, green channel and bright filed), Ld‐LPO (b, red channel, and bright field), merged image of panels a, b and bright field, and co‐localization curve of two dyes (Scale bar = 5 μm). (b) The confocal imaging results of lipid droplets stained with Ld‐LPO after 36 h in macrophages (e, red channel; f, green channel; g, merged image of panels e, f, and bright field; h, ratio image of e and f, Scale bar = 20 μm).

To validate the feasibility of Ld‐LPO in monitoring lipid droplet peroxidation (LPO), it is essential first to ensure its specific and stable localization within macrophage lipid droplets. Co‐staining of macrophages was conducted using Ld‐LPO and BODIPY493/503, the most commonly used lipid droplet tracer. Macrophages were sequentially stained with 5 μM Lp‐LPO and 1 μM BODIPY493/503 and imaged immediately. Fluorescence microscopy images of macrophages are shown in Figure 2a. The red fluorescence emitted by Ld‐LPO is distributed within small punctate organelles, which are also stained by the standard lipid droplet probe BODIPY493/503. The fluorescence images from both channels overlap remarkably well, with a Pearson correlation coefficient of 0.84, demonstrating satisfactory co‐localization (Figure 4d). These results confirm the specific targeting of macrophage lipid droplets by Ld‐LPO. Subsequent long‐term fluorescence tracking further confirmed the stable localization of Ld‐LPO within lipid droplets. After 36 h of Ld‐LPO staining (Figure 4e–g), macrophages maintained almost identical fluorescence intensity and cellular morphology. Additionally, Ld‐LPO could also target 4T1, HeLa, and liver cells, with sustained retention over the long term, indicating its applicability across multiple cell types. In conclusion, the stable targeting of lipid droplets by Ld‐LPO ensures its capability to continuously monitor changes in lipid droplet LPO levels during cellular damage. The cytotoxicity of Ld‐LPO is very low. MTT assays indicate that even with continuous culture of cells in 5 μM Ld‐LPO for 24 h, 95% of the cells remain viable. This highlights the advantage of LD‐LPO as a practical lipid droplet probe for use in live cells.

### 2.4 Evaluation of LPO in foam cells through fluorescence ratiometric imaging and flow cytometry study

**FIGURE 5:**
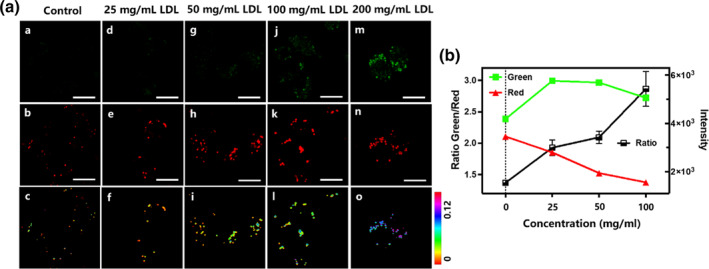
(a) Fluorescence images of stained (Ld‐LPO, 5 μM for 1 h) macrophage cells, incubated with ox‐LDL (20 mg/L) and different amounts of low‐density lipoprotein (LDL) (25/50/100/200 mg/L) for 36 h. (a, d, g, j, m, green channel; b, e, h, k, n, red channel; c, f, i, l, o, ratio images of green channel and red channel) Excitation wavelength: 488 nm. Green channel: 525 ± 25 nm. Red channel: 585 ± 25 nm (Scale bar = 20 μm). (b) Flow cytometry (FCM) result of stained (Ld‐LPO, 5 μM for 1 h) macrophage cells being incubated with ox‐LDL (20 mg/L) and different amounts of LDL (25/50/100/200 mg/L) for 36 h. Excitation wavelength: 488 nm. FITC‐A/Green channel: 530 ± 30 nm. PE‐A/Red channel: 585 ± 42 nm.

Due to the probe's excellent ability to target lipid droplets and its responsiveness to LPO in solution, we decided to use this probe for a systematic study of LPO changes in foam cells with varying levels of LPO.[^28^, ^29^] Macrophages were incubated for 36 h in different concentrations of low‐density lipoprotein (LDL) (25, 50, 100, 200 mg/L) along with a small amount of oxidized LDL (20 mg/L). Dual‐channel fluorescence images are presented in Figure 5a. The ratio imaging of cells under identical stimulation conditions was reproducible. Lipid droplets are visible in the fluorescence images, and significant spectral shifts are observed in these luminescent regions. As the dose of LDL increases, the fluorescence brightness of the green channel significantly increases. Such a notable spectral shift provides evidence of Ld‐LPO capturing LPO in lipid droplets. The Green/Red ratio exhibits a gradient increase, from 0.65 (50 mg/L LDL) to 1.44 (200 mg/L LDL), indicating a significant increase in the degree of LPO. However, these total ratios at different induction times only reflect the average LPO level of all lipid droplets at various stages of foam cell development. Fortunately, when overlapping the corresponding images of the two channels, it is observed that the green and red areas coincide entirely. This indicates that the green fluorescent products generated by the peroxidation of Ld‐LPO retain the same localization specificity as the initial probe for lysosomes. Additionally, many lipid droplets are inside the cells with the absolute number continuously rising. The lipid droplets are growing from the size of the luminescent contour. Overall, lipid droplets, during foam cell formation in macrophages, not only act as carriers for lipid transport but also undergo LPO simultaneously within them.

We attempted to utilize it in flow cytometry (FCM) to demonstrate the value of Ld‐LPO further. As anticipated, FCM analysis in the PE‐A channel (585 ± 42 nm) (Figure 5b) shows that the fluorescence signal in normal macrophages shifts from 4000 to 1500 units in foam cells. This indicates a significant reduction in intracellular fluorescence in foam cells, providing a fundamental and clear criterion for foam cell formation. On the other hand, dual‐color FCM analysis enables the relative quantification of LPO levels in foam cells within large cell populations. Macrophages were treated using the same method described for the cells in Figure 4a. The mean intensity values from both channels are plotted in Figure 5b. The average intensity plots for both channels (FITC‐A channel 530 ± 30 nm vs. PE‐A channel 585 ± 42 nm) reveal a noticeable trend. As the concentration of LDL increases, the signal intensity decreases in the red channel (PE‐A) while rising in the green channel (FITC‐A). The fluorescence intensity ratio between the two channels shows an apparent increase from approximately 1.5 to around 3.0. These FCM analyses provide intuitive and reliable information, and combining Ld‐LPO with FCM can distinguish foam cells from normal macrophages and quantify the LPO levels in foam cells within large cell populations.

### 2.5 Study of lipid droplet and lysosome interaction in foam cells

We know that a prominent characteristic of foam cells is the expansion of lysosomes, and it remains unclear whether they engulf lipid droplets during this lysosomal expansion or if the LPO level of lipid droplets is affected by lysosomal engulfment.^[1]^ As mentioned earlier, it is feasible to accurately assess the local LPO of individual lipid droplets by comparing the local Green/Red ratio. Therefore, we decided to use this probe to evaluate the changes in LPO levels of lipid droplets at different locations within cells.

**FIGURE 6:**
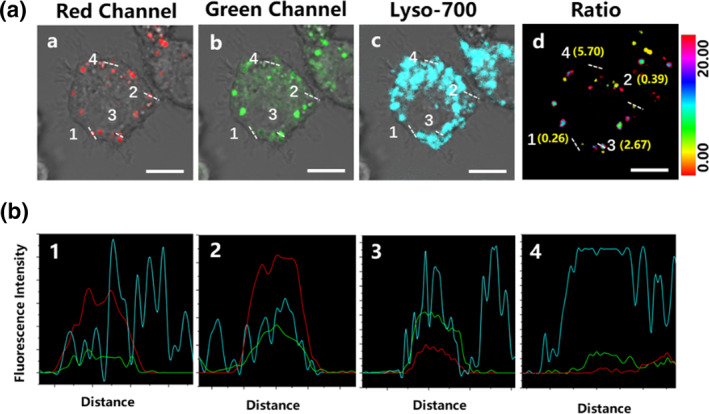
(A) Fluorescence images of foam Raw264.7 co‐stained by Ld‐LPO and Lyso‐700 (a, red channel and bright field, 570–630 nm; b, green channel and bright field, 500–550 nm; c, blue channel and bright field, 655–755 nm; d, ratio image of green channel and red channel) (Scale bar = 5 μm). (B) Co‐localization curves of Ld‐LPO (red channel and green channel) and Lyso‐700.

Macrophages were incubated with LDL (100 mg/L) and ox‐LDL (20 mg/L) for 36 h. The probe Ld‐LPO (5 μM for 1 h) was used for lipid droplet localization and LPO monitoring, while the lysosome probe Lyso‐700 (500 nM for 4 h) was utilized for foam cell lysosome localization. Three‐channel fluorescence images are presented in Figure 6. It can be observed that there are three types of lipid droplets within the foam cells. For the first type, the lipid droplet does not overlap with the lysosome, as seen in regions 1 and 2. Meanwhile, the fluorescence of Ld‐LPO in the red channel is significantly stronger than that in the green channel, with the green/red ratio (0.26 and 0.39) in Figure 6b1,b2 indicating a low LPO level. For the second type, the lipid droplet co‐localizes with the lysosome, as shown in region 3. Meanwhile, the fluorescence of Ld‐LPO is intense in both the red and green channels, with the green/red ratio (2.67) in Figure 6b3 indicating a moderate LPO level. For the third type, the lipid droplet co‐localizes with the lysosome, as shown in region 4. Meanwhile, the fluorescence of Ld‐LPO in the red channel is significantly weaker than that in the green channel, with the green/red ratio (5.70) in Figure 6b4 indicating a high LPO level. These results suggest that lysosomes in foam macrophages engulf lipid droplets, causing the probe within the lipid droplets to transfer into lysosomes. Therefore, Ld‐LPO overlapped with Lyso‐700 as the LPO proceeded. It is known that macrophages transform into foam cells after unlimited uptake of native LDL. As more native LDL accumulates inside the cells, the LPO process accelerates. This explains why the LPO level of engulfed lipid droplets is higher than that of unengulfed ones.

## 3 CONCLUSION

In summary, we developed a fluorescent LPO probe with lipid droplet targeting ability. Ld‐LPO can quantitatively evaluate the level of LPO in individual lipid droplets through ratiometric imaging and dual‐color FCM detection. By using Ld‐LPO, we found significant LPO in lipid droplets during the formation of foam cells. We also found that engulfed lipid droplets by lysosomes exhibit higher LPO. Consequently, we provide a smart probe for assessing LPO specifically within lipid droplets.

## 4 EXPERIMENTAL SECTION/METHODS

### 4.1 General methods

All chemicals were obtained from commercial suppliers and used without further purification. Melting points were determined using a melting point apparatus and uncorrected. ^1^H nuclear magnetic resonance spectroscopy (NMR) and ^13^C NMR were measured in CDCl_3_ with trimethylsilane (TMS) as internal reference. Coupling constants (J) are given in Hz. Column chromatography was performed with silica gel (200–300 mesh). All solvent mixtures are given as volume/volume ratios. Fluorescence quantum yields were determined using fluorescein as a reference.

### 4.2 Procedure for the synthesis of **Ld‐LPO**

**BDP** (381 mg, 1.18 mmol) and cinnamic aldehyde (297 μL, 2.36 mmol) were added to a 100 mL round‐bottomed flask containing 30 mL of toluene, and to this solution was added piperidine (1 mL) and acetic acid (1 mL). The mixture was heated under reflux using a Dean‐Stark trap, and the reaction was monitored by thin layer chromatography (TLC) 1:4 (v/v) CH_2_Cl_2_: Hexane (*R*_*f*_ = 0.3). When all the starting material has been consumed, the mixture is cooled to room temperature, and the solvent is evaporated. Water (300 mL) was added to the residue, and the product was extracted into the CH_2_Cl_2_ (3 × 100 mL). The organic phase was dried over Mg_2_SO_4_ and evaporated. The residue is purified by silica gel column chromatography using 1:4 CH_2_Cl_2_: Hexane as the eluent, which yielded the desired product **Ld‐LPO** as a purple solid (93 mg, 18%). Mp: 245–247°C. ^1^H NMR (CDCl_3_, 400 MHz): δ 7.50–7.44 (5H, m), 7.37–7.22 (6H, m), 7.14–7.03 (2H, m), 6.77–6.73 (1H, J = 16 Hz, d), 6.55 (1H, s), 6.01 (1H, s), 2.59 (3H, s), 1.41 (3H, s), 1.39 (3H, s). ^13^C NMR (CDCl_3_, 100 Hz): δ 155.5, 152.3, 142.9, 142.3, 140.2, 136.9, 136.7, 136.0, 135.1, 133.0, 132.0, 129.3, 129.1, 129.0, 128.8, 128.3, 128.2, 126.8, 123.1, 121.4, 117.8, 29.7, 29.3, 27.2, 22.7. m/z (ESI): Calcd [M + H]^+^ for C_28_H_26_BF_2_N_2_: 439.2079. Found: 439.2162.

### 4.3 Culture of RAW 264.7 and fluorescent imaging

RAW 264.7 (Macrophage cells) were obtained from the Institute of Basic Medical Sciences of the Chinese Academy of Medical Sciences. All cell lines were maintained under standard culture conditions (atmosphere of 5% CO_2_ and 95% air at 37°C) in RPMI 1640 medium supplemented with 10% FBS (fetal bovine serum).

Grow RAW 264.7 in the exponential growth phase on 35 mm glass‐bottom culture dishes (Φ 20 mm) for 1–2 days to reach 70%–90% confluency. These cells are used in colocalization and stimulation experimentation. For the co‐localization study, cells were washed with RPMI 1640 three times, and then incubated with 2 mL RPMI 1640 containing Ld‐LPO (5.0 μM) for 1 h and 2 mL RPMI 1640 containing BODIPY 493/503 (1.0 μM) for 5 min in turn at 37°C. The cells were washed twice with 1 mL PBS at room temperature, then 1 mL RPMI 1640 medium was added and observed under a confocal microscope (Olympus FV1000). For the stimulation study, macrophages in the exponential growth phase were plated into 35 mm glass‐bottom culture dishes (Φ 20 mm) containing 2 mL of RPMI 1640. After incubation at 37°C with 5% CO_2_ for 1–2 days, the media was removed to reach 70%–90% confluency. Then, the cells were washed with 2 mL of PBS buffer, and 2 mL of fresh RPMI 1640 containing LDL or ox‐LDL was added. Cells were stained sometime during the stimulation.

### 4.4 MTT assay

The cytotoxic effect of Ld‐LPO was assessed using the MTT assay. The cells in the exponential growth phase are used in the experimentation. 1.5 × 10^3^ cells/well are seeded onto 96‐well plates and allowed to grow for 24 h before treatment with Ld‐LPO. The incubation time of Ld‐LPO was 1 h with a concentration of 5 μM. At the end of this time, the Ld‐LPO containing medium is replaced with dye‐free medium. After 12 h or 24 h, MTT is added to each well (final concentration 0.5 mg/mL) for 4 h at 37°C, and formazan crystals formed through MTT metabolism by viable cells are dissolved in dimethyl sulfoxide (DMSO). Optical densities were measured at 490 nm.

### 4.5 Flow cytometry (FCM)

RAW 264.7 (Macrophage cells) were cultured in RPMI 1640 supplemented with 10% FBS (fetal bovine serum) in an atmosphere of 5% CO_2_ and 95% air at 37°C. For FCM studies, macrophages in the exponential growth phase were plated into 35 mm glass‐bottom culture dishes (Φ 20 mm) containing 2 mL of RPMI 1640. After incubation at 37°C with 5% CO_2_ for 1−2 days, the media was removed to reach 70%–90% confluency. Then, the cells were washed with 2 mL of PBS buffer, and 2 mL of fresh RPMI 1640 was added along with LDL or ox‐LDL. Cells were stained before stimulation. Samples were illuminated with a sapphire laser at 488 nm on a FACScan flow cytometer (BD Biosciences Pharmingen, USA). Each group of samples detected a total of 10,000 cells at 100 events per second. Analysis of FCM data with FlowJo software.

## CONFLICT OF INTEREST STATEMENT

The authors declare no conflicts of interest.

## ETHICS STATEMENT

No animal or human experiments were involved in this study.

## Supporting information

Supporting Information S1

## Data Availability

The data that support the findings of this study are available on request from the corresponding author. The data are not publicly available due to privacy or ethical restrictions.
