# Supplementary material for: A ratiometric fluorescent probe monitoring lipid peroxidation within lipid droplets in foam cells
Source: Smart Mol. 2025 Aug 19;3(3):e70015. doi: 10.1002/smo2.70015 (PMC12483125; doi:10.1002/smo2.70015)
Supplement: Supplementary file 1 — Supporting Information S1 [file SMO2-3-e70015-s001.docx]

A Ratiometric Fluorescent Probe Monitoring Lipid Peroxidation within Lipid Droplets in Foam Cells

Dingxuan Li ^1, #^, Caiwei Lu ^1, #^, Shulan Sun^2,^*, Xiaoxi Li^2^, Yi Xiao ^1,^* and Xinfu Zhang ^1,^*

^1^ State Key Laboratory of Fine Chemicals, Frontiers Science Center for Smart Materials Oriented Chemical Engineering, Dalian University of Technology, Dalian 116024, China.

^2^ Central Laboratory, Cancer Hospital of Dalian University of Technology (Liaoning Cancer Hospital & Institute), Dalian 116024, China.

E-mail: sunshulan@cancerhosp-ln-cmu.com; xiaoyi@dlut.edu.cn; zhangxinfu@dlut.edu.cn

**Supporting Information:**

**Supplemental Experimental Procedures**

1. General methods and materials

All chemicals were obtained from commercial suppliers and used without further purification. Melting points were determined using a melting point apparatus and uncorrected. ^1^H NMR and ^13^C NMR were measured in CDCl_3_ with TMS as internal reference. Coupling constants (*J*) are given in Hz. Column chromatography was performed with silica gel (200-300 mesh). Fluorescence quantum yields were determined using fluorescein as a reference.

1. The synthesis procedure of Ld-LPO

**Synthetic route:**

**BDP** (381 mg, 1.18 mmol) and cinnamic aldehyde (297 μL, 2.36 mmol) were added to a 100 mL round-bottomed flask containing 30 mL of toluene, and to this solution was added piperidine (1 mL) and acetic acid (1 mL). The mixture was heated under reflux using a Dean-Stark trap, and the reaction was monitored by TLC 1:4 (v/v) CH_2_Cl_2_: Hexane (R_f_ = 0.3). When all the starting material has been consumed, the mixture is cooled to room temperature, and the solvent is evaporated. Water (300 mL) is added to the residue, and the product is extracted into the CH_2_Cl_2_ (3×100 mL). The organic phase is dried over Mg_2_SO_4_ and evaporated. The residue is purified by silica gel column chromatography using 1:4 CH_2_Cl_2_: Hexane as the eluent, which yielded the desired product **Ld-LPO** as a purple solid (93 mg, 18%). Mp: 245−247°C. ^1^H NMR (CDCl_3_, 400 MHz): δ 7.50-7.44 (5H, m), 7.37-7.22 (6H, m), 7.14-7.03 (2H, m), 6.77 -6.73 (1H, J =16 Hz, d), 6.55 (1H, s), 6.01 (1H, s), 2.59 (3H, s), 1.41 (3H, s), 1.39 (3H, s). ^13^C NMR (CDCl_3_, 100 Hz): δ 155.5, 152.3, 142.9, 142.3, 140.2, 136.9, 136.7, 136.0, 135.1, 133.0, 132.0, 129.3, 129.1, 129.0, 128.8, 128.3, 128.2, 126.8, 123.1, 121.4, 117.8, 29.7, 29.3, 27.2, 22.7. m/z (ESI): Calcd [M+H]^+^ for C_28_H_26_BF_2_N_2_: 439.2079. Found: 439.2162.

1. **Common lipophilic dyes and their corresponding ClogP values**

The ClogP value is the LogP value of a compound predicted by computer simulation, representing the expected lipophilicity of the compound. Using ChemDraw 20 for the lipophilicity prediction of compound Nile Red, BODIPY 493/503, and LD-LPO.

**Figure S1**. The structures of common lipophilic dyes and Ld-LPO.

1. **Culture of cells and fluorescent imaging**

Culture of RAW 264.7 and Fluorescent Imaging RAW 264.7 (Macrophage cells) are obtained from the Institute of Basic Medical Sciences (IBMS) of the Chinese Academy of Medical Sciences (CAMS). All cell lines are maintained under standard culture conditions (atmosphere of 5% CO_2_ and 95% air at 37 °C) in RPMI 1640 medium, supplemented with 10% FBS (fetal bovine serum).

Grow RAW 264.7 in the exponential growth phase on 35 mm glass-bottom culture dishes (Φ 20 mm) for 1−2 days to reach 70%−90% confluency. These cells are used in colocalization and stimulation experimentation. For the co-localization study, cells were washed with RPMI 1640 three times, and then incubated with 2 mL RPMI 1640 containing Ld-LPO (5.0 μM) for 1 h and 2 mL RPMI 1640 containing BODIPY 493/503 (1.0 μM) for 5 minutes in turn at 37 ℃. The cells were washed twice with 1 ml PBS at room temperature, then 1 ml RPMI 1640 medium was added and observed under a confocal microscope (Olympus FV1000). For the stimulation study, macrophages in the exponential growth phase were plated into 35 mm glass-bottom culture dishes (Φ 20 mm) containing 2 mL of RPMI 1640. The media was removed after incubation at 37 °C with 5% CO_2_ for 1−2 days to reach 70%−90% confluency. Then the cells were washed with 2 mL of PBS buffer, and 2 mL of fresh RPMI 1640 containing LDL or ox-LDL was added. Cells were stained sometime during the stimulation.

We also conducted a co-localization experiment of LD-LPO in HL7702 cells, which can prove the universality of this probe.


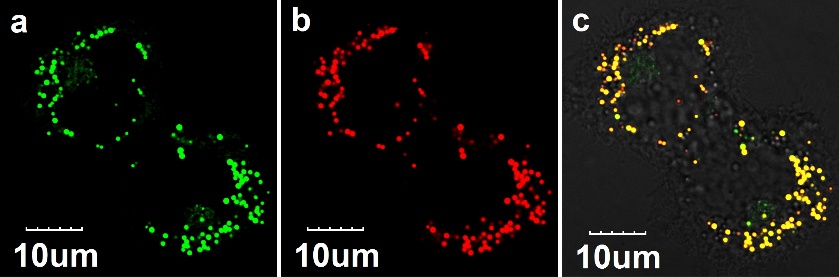


**Figure S2.** Fluorescence images of HL7702 cells co-stained by BODIPY 493/503 (a, green channel, λ_ex_ = 488 nm, λ_em_ = 500-550 nm) and LD-LPO (b, red channel, λ_ex_ = 488 nm, λ_em_ = 570-630 nm). (c) Merged image of a, b, and bright field. (d) Fluorescence image of stained HL7702 cells after incubation in standard culture medium for 24 h.

Co-localization imaging experiment proves LD-LPO is specific to lipid droplets in living cells. HL7702 cells were first incubated in culture medium containing fat emulsion (2 mL/L) for 48 h. When lipid droplets form on a large scale, cells are believed to have turned into fatty liver cells. These cells are co-stained with 1 μM LD-LPO and 1 μM BODIPY493/503(commercial probe for lipid droplet) for 1 h. Figure 2 shows a perfect colocalization result with an overlap coefficient of up to 0.97. Both channels show intensive and uniform lipid droplets without any background fluorescence. In addition, Figure 2d is the fluorescent image of stained fatty liver cells after incubation for another 24 h in standard culture medium. The photo shows clear lipid droplets with intense red fluorescence, which means LD-LPO can stay stably in lipid droplets. It’s essential for the detection of LPO that proceeds over a relatively long time. These results indicate LD-LPO can be used to study LPO in lipid droplets in fatty liver cells.

**MTT Assay:**

The cytotoxic effect of Ld-LPO is assessed using the MTT assay. The cells in the exponential growth phase are used in the experimentation. 1.5 × 10^3^ cells/well are seeded onto 96-well plates and allowed to grow for 24 h before treatment with Ld-LPO. The incubation time of Ld-LPO is 1 h with a concentration of 5 μM. At the end of this time, the Ld-LPO-containing medium is replaced with dye-free medium. After 12 h or 24 h, MTT is added to each well (final concentration 0.5 mg/mL) for 4 h at 37 °C, and formazan crystals formed through MTT metabolism by viable cells are dissolved in DMSO. Optical densities are measured at 490 nm.

**Figure S3**. Viability of cells with **Ld-LPO** (1 μM) after treatment for 12 h and 24 h.

**Flow Cytometry (FCM):**

RAW 264.7 (Macrophage cells) were cultured in RPMI 1640 supplemented with 10% FBS (fetal bovine serum) in an atmosphere of 5% CO_2_ and 95% air at 37 °C. For FCM studies, macrophages in the exponential growth phase were plated into 35 mm glass-bottom culture dishes (Φ 20 mm) containing 2 mL of RPMI 1640. The media was removed after incubation at 37 °C with 5% CO_2_ for 1−2 days to reach 70%−90% confluency. Then the cells were washed with 2 mL of PBS buffer, and 2 mL of fresh RPMI 1640 was added along with LDL or ox-LDL. Cells are stained before stimulation. Samples were illuminated with a sapphire laser at 488 nm on a FACScan flow cytometer (BD Biosciences Pharmingen, USA). Each group of samples detected 10,000 cells at 100 events per second—flow cytometry data analysis with FlowJo software.

1. **^1^H, ^13^C NMR Spectra of Ld-LPO**

**Figure S4.** ^1^H NMR Spectra of Ld-LPO

**Figure S5.** ^13^C NMR Spectra of Ld-LPO
